# Supplementary material for: Sn catalyst reconstruction and microenvironment modulation for efficient amino acid electrosynthesis via C–N coupling
Source: Nat Commun. 2026 Apr 21;17:3614. doi: 10.1038/s41467-026-71694-4 (PMC13100173; doi:10.1038/s41467-026-71694-4)
Supplement: Supplementary file 1 — Supplementary Information [file 41467_2026_71694_MOESM1_ESM.docx]

Supplementary Information

**Sn catalyst reconstruction and microenvironment modulation for efficient amino acid electrosynthesis via C**–**N coupling**

Han, S. *et al.*

**Supplementary Methods**

**Techno-economic Analysis (TEA).^1^** TEA was conducted based on a recently published model. The specific parameters calculated are based on our experimental results. Notably, this is just a simplified calculation model for easy comparison with other reports. Actual industrial production may differ significantly. The costs of the handling of residual reactants, NOx management, and catalyst degradation et. al, which may be involved in practical applications, are not considered in this paper due to the lack of actual industrial application parameters.

Some basic assumptions are as follows:

1. Operating condition: Current density was 1 A cm^-2^. Voltage was −1.4 V vs. SHE. FE of glycine was 90%. Daily production was 50 ton of glycine.
2. The price of OA was 313.18 USD ton^-1^. The price of HNO_3_ (68 wt%) was 225.1 USD ton^-1^. The price of H_2_O was 0.7 USD ton^-1^. The price of glycine was 2060.44 USD ton^-1^, all the cost of reactants come from the https://www.100ppi.com and the published literature. The stack cost was set as 275.55 USD kW^-1^. The electricity price was 0.05 USD kWh^-1^.
3. The expected service life of the electrolyzer was 15 years, with a working duration of 350 days per year.
4. The electrolyzer cost per area was calculated as follows:

Electrolyzer cost per area = $275.55 USD {kW}^{-1} \times\frac{0.9A}{{cm}^{2}} \times1.4 V \times\frac{{10}^{4} {cm}^{2}}{m^{2}} \times\frac{kW}{1000 W}=4860.7 USD m^{-2}$.

(5) The cost of Balance of plant was estimated to be 58% of the cost of the electrolyzer.

(6) The maintenance cost was determined to be 2.5% of the electrolyzer cost.

(7) Since no impurity salts are added to our system, the product can be separated by evaporation. Therefore, the separation cost is negligible.

(8) The relevant paraments were calculated as:

𝑇𝑜𝑡𝑎𝑙 𝑐𝑢𝑟𝑟𝑒𝑛𝑡 = $\frac{50000kg}{day} \times\frac{1000}{kg} \times\frac{mol}{75.07g} \times12e \times\frac{96485 C}{mol} \times\frac{day}{86400s}=8925465.86 A$

𝐸𝑙𝑒𝑐𝑡𝑟𝑜𝑙𝑦𝑧𝑒𝑟 𝑎𝑟𝑒𝑎 = $8925465.86 A\times\frac{m^{2}}{0.9 A} \times\frac{m^{2}}{{10}^{4} {cm}^{2}} =991.72 m^{2}$

𝑃𝑜𝑤𝑒𝑟 = $1.4 V\times8925465.86 A \times\frac{W}{{10}^{6} MW} =12.49 MW$

**Capital cost:**

1. Electrolyzer:

𝐸𝑙𝑒𝑐𝑡𝑟𝑜𝑙𝑦𝑧𝑒𝑟 𝑐𝑜𝑠𝑡 𝑝𝑒𝑟 𝑡𝑜𝑛 𝑔𝑙𝑦𝑐𝑖𝑛𝑒 =$4860.7 USD\times991.72 m^{2} \times\frac{1}{15 year} \times\frac{year}{365 day} \times\frac{day}{50 ton} =17.61 USD {ton}^{-1}$

1. Balance of plant:

Balance of plant per ton glycine = 17.61 USD x 0.58 = 10.21 USD ton^-1^

Operating cost:

1. Electricity:

Electricity cost = $12.49 MW\times\frac{1000 W}{MW} \times\frac{24 h}{day} \times0.05 \times\frac{USD}{kWh} \times\frac{day}{50 ton}=299.76 USD {ton}^{-1}$

1. Maintenance:

Maintenance cost = 17.61 USD ton-1 x 0.025 = 0.44 USD ton^-1^

**Materical cost:**

Based on the experimental results, the production of one ton of glycine requires 1.2 tons of OA, 1.235 tons of nitric acid (68%), and 29 tons of water.

𝑀𝑎𝑡𝑒𝑟𝑖𝑎𝑙 𝑐𝑜𝑠𝑡 = 1.2 ton x 313.18 USD ton^-1^ + 1.235 ton x 225.1 USD ton^-1^ + 29 ton x 0.7 USD ton^-1^ = 674.114 USD ton^-1^

**Total cost:**

Total cost = 17.61 + 10.21 + 299.76 + 0.44 + 674.11 = 1002.13 USD ton^-1^

**Supplementary Note 1**

The process of electrosynthesis of glycine can be understood as the coupling of nitrate reduction products and OA reduction products. Recent reports show that the products of nitrate reduction have NO_2_, N_2_, NH_2_OH, and NH_3_, and the products of OA electroreduction are glyoxylic acid (GOA) and glycolic acid (GA). According to classical organic chemistry, NH_2_OH will spontaneously couple with aldehyde group (−C=O) to form oxime (−C=N−OH). The C=N of oxime is an unsaturated bond and can be further hydrogenated to −C−N−. Therefore, the total reaction can be considered as a coupling of electrochemical-chemical processes, and is divided into four parts (Figure 1a). In this synthetic route, the most critical factor is to control the semi-hydrogenation of the two reactants (most of reports for nitrate reduction or OA reduction are fully hydrogenated products, ammonia or GA). Although a few paper references to semi-hydrogenation are available, the complex synthetic methods or highly toxic heavy metal elements make them difficult to be promoted. Therefore, before the co-reduction test, we first screened suitable candidate elements that could satisfy the above sub-reactions (R1 to R4). Notably, the catalyst we ultimately selected is based on its ability to perform the above sub-reactions, so the catalyst obtained by this screening method will also follow the above path in the overall reaction process (co-reduction system). However, this screening method cannot exclude other effective elements that can catalyze the overall reaction, because there may be many mechanisms for the electrosynthesis of Gly.

**Supplementary Table 1 | Screening of catalysts.** Screen for suitable catalyst elements capable of simultaneously facilitating the three sub-reactions.

| **Catalyst Element** | **FE of R1 (%)** | **FE of R2 (%)** | **FE of R4 (%)** |
| --- | --- | --- | --- |
| Fe | 10.37746 | 0 | 58.07044 |
| Co | 0 | 0 | 65.77039 |
| Ni | 14.74223 | 0 | 51.17257 |
| Cu | 2.86738 | 0 | 55.50379 |
| Zn | 3.41246 | 3.81504 | 96.42016 |
| Ag | 17.77085 | 0 | 82.77444 |
| Au | 8.299 | 0 | 83.89735 |
| **Sn** | **21.73162** | **65.13183** | **89.19106** |

Sub-reaction R1, R2 and R4 correspond to those shown in Figure 1a. R3 is a spontaneous reaction under ambient conditions and does not require any electrocatalyst.

The screening conditions are chronopotentiometry at −100 mA cm^−2^, electrode area of ​​1 cm^2^, and running time of 1 hour.

**Supplementary Note 2**

According to Figure 1b, these catalysts all showed the performance of synthesizing glycine at pH~0.4, except Co. However, the dissolution of Fe, Ni and Zn clearly suggested the instability of their structure. The prices of Ag and Au are expensive, which limits their production applications. Figure 1c showed that Cu exhibited poor FE for each sub-reaction, suggesting that it is not a good candidate to obtain glycine using our strategy. Therefore, we chose Sn as a model catalyst for subsequent experiments and mechanism exploration.

**Supplementary Figure** **1** | **XRD spectra.** The XRD spectra of CP **(a)** and Sn foil **(b)**, respectively.

**Supplementary Figure 2** | **Standard curves of C**−**N products quantification via the ^1^H NMR.** ^1^H NMR spectra with different concentration of glycine (Gly, **a**), glyoxylic acid oxime (GAO, **b**), and oxamic acid (OMA, **c**), respectively. The corresponding standard curve of Gly (**d**), GAO (**e**), and OMA (**f**), respectively. The calibration curve shows good linearity.

**Supplementary Figure 3** | **Standard curves quantification of C− or N− byproducts via the ^1^H NMR. a,** ^1^H NMR spectra with different concentration of NH_4_^+^, glyoxylic acid (GOA). The corresponding standard curve of NH_4_^+^ (**b**), and GOA (**c**), respectively. ^1^H NMR spectra with different concentration of glycolic acid (GA, **d**), ethylene glycol (**e**), and glyoxal (**f**), respectively. The corresponding standard curve of GA (**g**), ethylene glycol (**h**), and glyoxal (**i**), respectively. The calibration curve shows good linearity.

**Supplementary Note 3**

Quantitative analysis of hydroxylamine: Since hydroxylamine has no characteristic peaks in ^1^H NMR, its quantitative calculation using ^1^H NMR is based on the standard curve of GAO (Supplementary Figure 3b and 3e). Specifically, we would test two samples in parallel for the same testing solution, named sample A and B. Sample A contains 0.5 mL electrolyte, 50 μL DMSO-d_6_ and 50 μL deionized water. This sample can be used to quantify all products except hydroxylamine. Sample B contains 0.5 mL electrolyte, 50 μL DMSO-d_6_ and 50 μL GOA. Due to the aldehyde group and hydroxylamine can spontaneously generate oxime (R3 in Figure 1a). This reaction is very fast and thorough. Moreover, excess glyoxylic acid can ensure that the hydroxylamine in the test solution is almost fully reacted. Finally, the difference in the concentration of GAO between samples A and B is the hydroxylamine concentration.


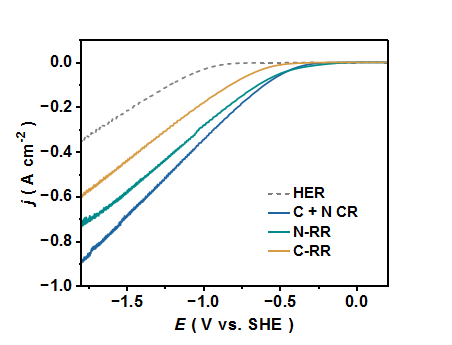


**Supplementary Figure 4** | **Electrocatalytic LSV curves.** LSV curves for the HER, OA electroreduction (C-RR), HNO_3_ electroreduction (N-RR), and OA + HNO_3_ co-electroreduction (C+N RR) over Sn nanoparticles under 1,600 r.p.m. without iR correction.


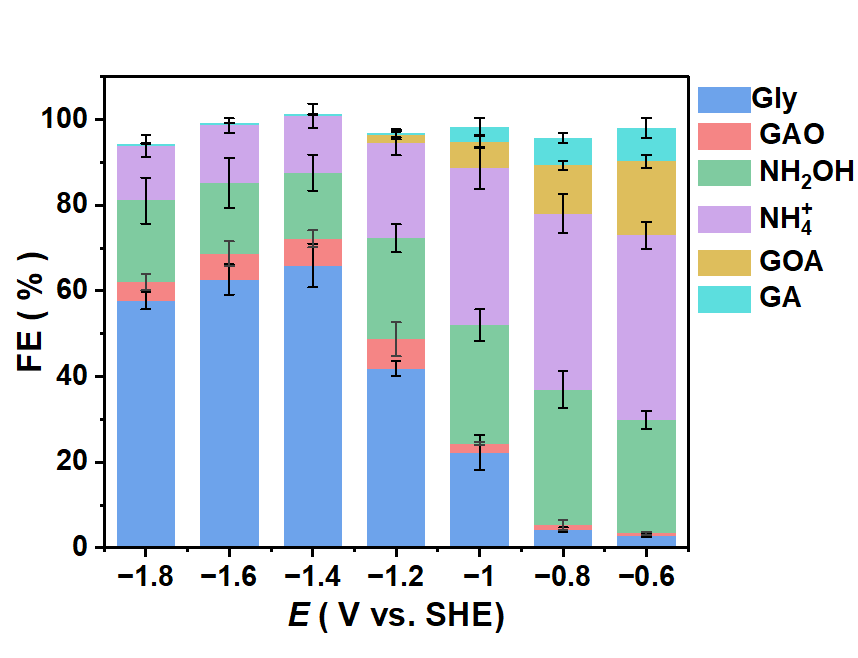


**Supplementary Figure 5** | **Potential-dependent FE of all products.** All products were detected by 1H NMR and their concentrations were calculated using the corresponding standard curves.


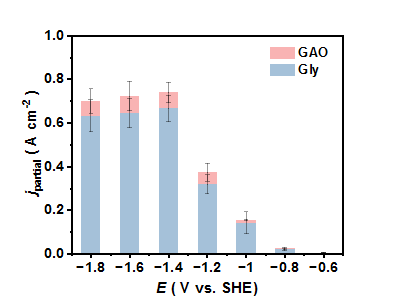


**Supplementary Figure 6** | **Electrocatalytic performance for the Gly synthesis.** Partial current density of C−N coupling products including Gly and GAO. The electrolyte is 0.5 M HNO_3_ + 0.5 M OA and the volume is 8 mL.

**Supplementary Table 2 | Summary of the electrocatalytic performance in C**−**N coupling for amino acids synthesis.**

| **No.** | **Reactant** | | **Catalyst** | **Optimal potential / V vs. SHE** | **FE / %** | **Yield rate / mmol cm^−2^ h^−1^** | **Ref.** |
| --- | --- | --- | --- | --- | --- | --- | --- |
|  | **N−source** | **C**−**source** |  |  |  |  |  |
| 1 | HNO_3_  0.5 M | OA  0.5 M | amorphous-Sn | −1.4 | 65.9 | 2.07 | This work |
| 2 | HNO_3_  0.25 M | OA  0.25 M |  | −1.4 | 73.8 | 1.60 |  |
| 3 | HNO_3_  0.1 M | OA  0.1 M |  | −1.4 | 56.8 | 0.79 |  |
| 4^a^ | HNO_3_  0.5 M | OA  0.5 M |  | / | 93 | 2.91 |  |
| 5^b^ | NaNO_3_  0.5 M | OA  0.5 M | Single atom Fe-N-C | −1.3 | 64.2 | 0.33 | ^2^ |
| 6 | NaNO_3_  0.25 M | OA  0.25 M | Cu-Hg | −1.6 | 26.0 | 0.28 | ^3^ |
| 7 | KNO_3_  0.3 M | GOA  0.3 M | CuBi-MOF | / | 65.9 | 0.15 | ^4^ |
| 8 | NaNO_3_  1 M | GOA  0.1 M | Fe-TiO_2_ | −0.7 | / | 0.24 | ^5^ |
| 9 | NaNO_3_  0.5 M | OA  0.5 M | Pb | / | <15 | / | ^6^ |
| 10^c^ | NaNO_3_  0.5 M | OA  0.5 M | Pb | / | 59 | 0.82 | ^6^ |
| 11 | NaNO_3_  0.5 M | OA  0.5 M | Bi | −0.86 | 75.9 | 0.212^b^ | ^7^ |
| 12 | NaNO_3_  0.5 M | OA  0.5 M | IL@Bi | −1.3 | 81.1 | 0.721^b^ | ^1^ |
| 13 | NaNO_3_  0.25M | OA  0.25M | PbSnBi | −1.5 | 57.2 | 1.12 | ^8^ |
| 14 | NaNO_3_  0.075M | OA  0.16M | TiO_2_ | −0.7 | 36 | 0.049 | ^9^ |
| 11 | NO | Pyruvate | Ag | −0.97 | 17 | 0.011 | ^10^ |
| 12^c^ | NO | Pyruvate | Ag | / | 70 | 0.37 | ^10^ |
| 13 | NO | Pyruvate | Single atom Fe | −0.65 | / | 0.032 | ^11^ |

^a^The experimental data is derived from a scale-up flow-cell, which is a two-electrode system without a reference electrode.

^b^Some data are derived from calculation results according to the data published in their paper.

^c^The experiment adopted a two-step tandem approach rather than a one-pot method.

According to the recent reports, it can be seen that high FE usually corresponds to low yield rate. And high yield rate is achieved in a system where multiple steps are connected in series. Our work can achieve both high FE and yield rate in a one-pot reaction, suggesting its advanced catalytic properties. Notably, Sn may also be a good candidate for some recent researches on the synthesis of non-conjugated oximes via C−N coupling, because this step is included in our system.

**Supplementary Figure 7 | ^1^H NMR spectra of synthetic Gly using different N−reactant.** The ^1^H NMR spectra for electrolyte with different N−reactants after electrocatalytic testing. Using KNO_3_ (**a**), KNO_2_ (**b**), and NO gas (**c**) to replace the HNO_3_ in original reaction system (Figure 2b). Here, the pH of the electrolyte is regulated by 0.25 M H_2_SO_4_ solution. The other conditions were the same as Figure 2b. The characteristic peaks marked by pink boxes represent the GAO, and the blue boxes represent the Gly. Supplementary Figure 6a showed a same result as Figure 2b, which proves that the addition of K^+^ would not affect this reaction. A possible important phenomenon for using KNO_2_ as a N−reactant is that a large amount of bubbles can be generated after adding KNO_2_ into the acidic electrolyte. This may be the NO_2_^−^ decomposition process, which can reduce the NO_2_^−^ concentration and thus the glycine yield is lower. Adding KNO_2_ in multiple times can effectively improve the synthesizing glycine performance.

**Supplemetary Note 4**

Nitrite are unstable in acidic solutions and spontaneously undergo a disproportionation reaction to generate NOx gas, which directly results in the actual N-reactant being far lower than our expected input. Here, we use a multiple addition method to test the co-reduction performance. We divide KNO_2_ into 6 equal portions and add one portion to the electrolyte every 20 minutes.

**Supplementary Figure 8** | **^1^H NMR spectra of C**−**N coupling reaction using different C−reactant.** The ^1^H NMR spectra for electrolyte with different C−reactants after electrocatalytic testing. Using formic acid (**a**), acetic acid (**b**), benzoic acid (**c**) and succinic acid (**d**) to replace the OA in original reaction system (Figure 2b). The other conditions were the same as Figure 2b. The red boxes indicate the characteristic peaks of the reactants, and The orange boxes indicate the characteristic peak of by-product NH_4_^+^. The results showed that no corresponding oxime and amine were generated.

**Supplementary Figure 9** | **^1^H NMR spectra of individual carboxylic acid reduction reaction.** The ^1^H NMR spectra for the control experiments in Supplementary Figure 7 without adding N-reactant. Using 0.5 M formic acid (**a**), acetic acid (**b**), benzoic acid (**c**) and succinic acid (**d**) and 0.25 M H_2_SO_4_ as an electrolyte, respectively. The other conditions were the same as Figure 2b. The red boxes indicate the characteristic peaks of the reactants. The results showed that no corresponding aldehyde was generated.

**Supplementary Figure 10** | **^1^H NMR spectra of C**−**N coupling reaction using different C−reactant.** The ^1^H NMR spectra of electrolyte after electrocatalytic C−N testing with different C−reactants. Using formaldehyde (**a**), acetaldehyde (**b**), and acetone (**c**) to replace the OA in original reaction system (Figure 2b). The other conditions were the same as Figure 2b. The characteristic peaks marked by red boxes represent the formaldehyde oxime (in Supplementary Figure 9a), acetaldehyde oxime (in Supplementary Figure 9b), acetone oxime (in Supplementary Figure 9c), respectively. The illustrations show their molecular structures. These oximes were quantified according to their corresponding standard curves.

**Supplementary Figure 11** | **^1^H NMR spectra of the synthesis of amino acids.** The ^1^H NMR spectra for electrolyte after electrocatalytic testing using different water-soluble *α*-carbonyl acids as reactants. Using GOA (**a**), pyruvate (**b**), and *α*-ketoglutarate (**c**) to replace the OA in original reaction system (Figure 2b). The other conditions were the same as Figure 2b. The characteristic peaks marked by red boxes represent the GAO (Supplementary Figure 10a), pyruvate oxime (Supplementary Figure 10b), ketoglutarate oxime (Supplementary Figure 10c), respectively. The illustrations with red background are their molecular structure. The blue boxes represent the Gly (Supplementary Figure 10a), alanine (Supplementary Figure 10b), and glutamate (Supplementary Figure 10c), respectively. The illustrations with blue background are their molecular structure. These oximes and amino acids were quantified according to their corresponding standard curves.

**Supplementary Note 5**

Define chain reaction and direct interfacial hydrogenation reaction in this work:

Chain reaction:

According to figure 2a, the synthesis process of Gly requires multiple reaction steps and multiple key intermediates. Some intermediates are both products of the previous reaction and reactants of the next reaction. During the electrocatalytic process, these intermediates are desorbed into the electrolyte as products. When the concentration accumulates to a certain amount, it returns to the electrode surface as a reactant for the next reaction step. Thus, similar to the domino effect, the appearance of intermediates has a clear order, and their concentrations need to accumulate. The FE of the final product continues to increase from 0 over time. During a long reaction process, the concentration of dual-identity intermediate will gradually stabilize, which is a balance between its generation rate and consumption rate. The disadvantage of this mechanism is that it is difficult to obtain a high proportion of the final product in a short period of time. And, the final products and intermediates coexist in the system, which leads to a low FE of final product Gly.

Interfacial hydrogenation reaction:

The difference from the above mechanism is that the reactants conversion process takes place on the catalyst surface, and the key intermediate would not be desorbed as a previous-step product until they are converted into the final product. It is a frequently reported mechanism in the electrocatalysis field recently. The fundamental reason is the difference in the adsorption and desorption energies of the intermediates. In this mechanism, the generated intermediates do not desorb but continue to proceed to the subsequent reaction steps. The advantage of this mechanism is that the final product can be obtained directly and the final product FE is stable (at a constant potential). If there are no other side reactions interfering in the system, it can achieve high FE at any stage of the reaction process. Similar to the bottleneck effect, this process may be affected by the rate-limiting step, resulting in a low overall reaction rate.

Notably, the above explanation is an ideal situation. In actual catalytic processes, the two mechanisms may coexist, or different mechanisms may be exhibited for different reactants. For example, in our system, the key intermediate NH_2_OH is desorbable in both mechanisms. In this work, the judgment of these two mechanisms is based on the evolutionary process of C−species, because the conversion of C-species is the rate-limiting step in the synthesis of Gly (evidence will be provided below). Considering the limitations of experimental detection sensitivity, we distinguish between the two mechanisms here based on the NMR results of the intermediate concentration. When the desorption amount of GOA and GAO intermediates is below the NMR detection limit, we consider it to have no apparent desorption behavior. The C-N coupling process likely involves the desorbed NH₂OH acting as a nucleophile to couple with the adsorbed *GOA at the Sn surface, transforming it into *GAO. There may be other complex processes involved, but since these cannot be determined experimentally, we will not speculate further.

**Supplementary Figure 12** | **Time-dependent products monitoring.** Concentration changes (**a**) and partial currents density (**b**) of different products. The grey line in Supplementary Figure 11b is the total current density. The concentration change (Δ*c*) is the difference between two adjacent test results. The partial current density is calculated based on the change in concentration and the number of electrons transferred during each time period. The error bars for the x values ​​represent the time period of the data. Among them, the electron transfer number of Gly, GAO, OMA, NH_2_OH, NH_4_^+^, GOA and GA are 12, 10, 10, 6, 8, 2 and 4, respectively. The error bars for the y values (wide ribbon) correspond to the standard deviations of three independent measurements, and the centre value for the error bars is the average of the three independent measurements. The error bars for the y values (wide ribbon) correspond to the standard deviations of three independent measurements, and the centre value for the error bars is the average of the three independent measurements.

**Supplementary Note 6**

During the whole process, the changes of the reactant concentration is negligible (from 0.5 M to >0.4 M). Therefore, the reactant concentration can be considered as a steady state. According to the classic chain reaction law, as the reaction continues, the concentration of the intermediate products (NH_2_OH and GOA) should tend to be stable (the generation and consumption rates reach a balance). At the beginning of the reaction, the intermediate such as GOA accumulates, which due to the generation rate of GOA is greater than the consumption rate. As the concentration of GOA increases, the consumption rate will also increase until the consumption rate and generation rate reach a balance, consisting with the chain reaction mechanism described above. However, after half an hour, its concentration did not maintain stable, but gradually decreased, corresponding to the consumption rate being greater than the generation rate. Since GAO and Gly continue to be produced, and FE and concentration of Gly showed an upward trend. This means that GOA is still the key intermediate, but it cannot be accumulated in the electrolyte. Therefore, there may be other mechanisms interfering with the initial chain reaction process, which may go through the GOA intermediates (to ensure the generation of GAO intermediates), but will not produce free/desorbed GOA molecules. A similar phenomenon can also be observed for the GAO yield rate about 1 hour. To distinguish it from a chain reaction, we refer to this process of conversion at the interface as the direct interface hydrogenation mechanism. In this process, the reactant molecule (OA) conversion always occurs on the catalyst interface and is not desorbed until it transforms into Gly.

**Supplementary Figure 13** | **The performance of OMA electroreduction.** ^1^H NMR tests for the 500 μL electrolytes + 50 μL DMSO−d_6_ and 50 μL DI water after 2h electro-testing at −1.4V vs. SHE. The initial electrolyte is 0.5 M OMA + 0.25 M H_2_SO_4_. The table is a summary of the molecules represented by different peaks (marked by cyan frame) in the left ^1^H NMR spectra. The results showed that the yield of Gly was extremely low, proving that the conversion pathway from OMA to Gly could be ignored. The NH_4_^+^ may come from the hydrolysis reaction of OMA in acidic solution (OMA + H_2_O + H^+^ → NH_4_^+^ + OA), and the GOA and GA may be the reduction product of OA.

**Supplementary Figure 14** | **SEM and ICP−OES testing.** (**a**) SEM image of Sn catalyst after electro-testing. Compared with Figure 1a, the morphology of the catalyst has no obvious change, suggesting that the catalyst is stable and has no obvious shedding or dissolution phenomenon. (**b**) Monitoring of Sn ions in electrolyte for different reaction times, eliminated Sn dissolution.

**Supplementary Figure 15 | The structural characterization of Sn catalyst after electro-testing. (a)** HR−STEM image of Sn nanoparticles after electro-testing. Compared with Figure 1e, the high atomic disorder indicates an amorphous structure. (**b**) XRD pattern of self-supported Sn nanoparticles loaded on C paper after electro-testing.

**Supplementary Figure 16 | CV test for oxidation−reduction peaks.** CV curves of SnO_2_ (**a**) and SnO (**b**) standard samples. The electrolyte solution is 0.25 M H_2_SO_4_. The test results showed that the electroreduction peak of SnO and SnO_2_ were −0.31 V vs. SHE and + 0.59 V vs. SHE, respectively.


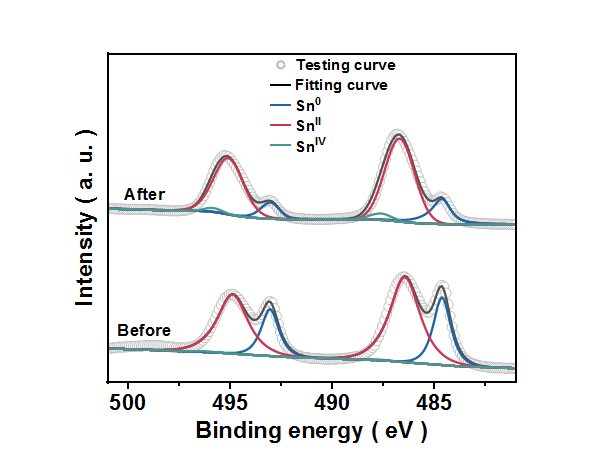


**Supplementary Figure 17 | XPS spectra of Sn.** XPS spectra of Sn before and after electro-testing. After test, the proportion of Sn^0^ decreased significantly. There are two possibilities. One is that the valence state of Sn^0^ may be in situ converted to SnO during the electrocatalytic process. The second possibility is that the surface atoms of amorphous-Sn are more active, which can be more easily oxidized in the air and becomes SnO.


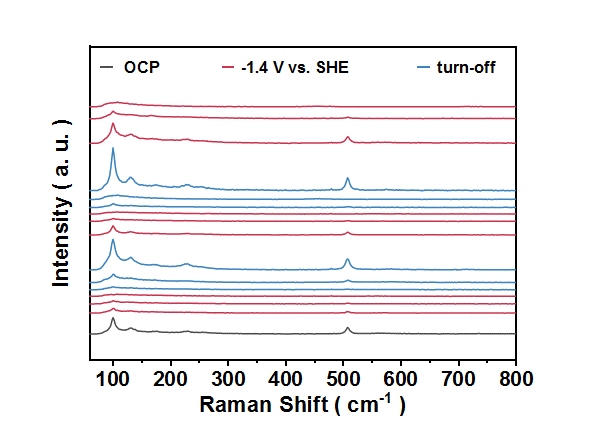


**Supplementary Figure 18 | The intermittent in situ Raman test.** The grey lines are collected at open circuit potential (OCP) over Sn nanoparticles. The red lines are collected at –1.4 V vs. SHE and the blue lines are collected without any potential. Freshly prepared Sn nanoparticles are selected as catalysts. The electrolyte is 0.5 M HNO_3_ + OA mixed solution. The appearance and disappearance of the characteristic peak represent the continuous proceeding of the redox reaction (Eq. 1) and the electroreduction reaction (Eq. 2). Combined with the DEMS results (Figure 4f), the specific equations as followed:

2NO_3_^−^ + 3Sn + 2H^+^ → 2NO + 3SnO + H_2_O (1)

SnO +2e^−^ + H+ → Sn + H_2_O (2)

**Supplementary Figure 19 | In situ Raman measurement of Sn in 0.5 M OA + 0.25 M H_2_SO_4_ electrolyte.** (**a**) In situ Raman tests for OA electroreduction over Sn. The experimental potential is form 0 V to −1.8 V vs. SHE. (**b**) The intermittent in situ Raman test for OA electroreduction over Sn. The grey lines are collected at open circuit potential (OCP) over Sn nanoparticles. The red lines are collected at –1.4 V vs. SHE and the blue lines are collected without any potential. (**c**) Heatmap for intermittent in situ Raman measurements, the color bar range is the same as Figure 4c, and dark red represents strong absorption intensity. Compared with Figure 4c, Supplementary Figure 20c shows main blue and faint yellow belts, suggesting weak characteristic peaks.

**Supplementary Figure 20 | In situ Raman measurement of Sn in 0.5 M HNO_3_ electrolyte.** (**a**) In situ Raman tests for HNO_3_ electroreduction. The experimental potential is form 0 V to −1.8 V vs. SHE. (**b**) The intermittent in situ Raman test for HNO_3_ electroreduction. The grey lines are collected at open circuit potential (OCP) over Sn nanoparticles. The red lines are collected at –1.4 V vs. SHE and the blue lines are collected without any potential. (**c**) Heatmap for intermittent in situ Raman measurements. The color bar range is the same as Figure 4c, and dark red represents strong absorption intensity. Compared with Figure 4c and Supplementary Figure 20c, Supplementary Figure 21c shows same red belts, which suggest that the spontaneous redox process is related to reactant HNO_3_.


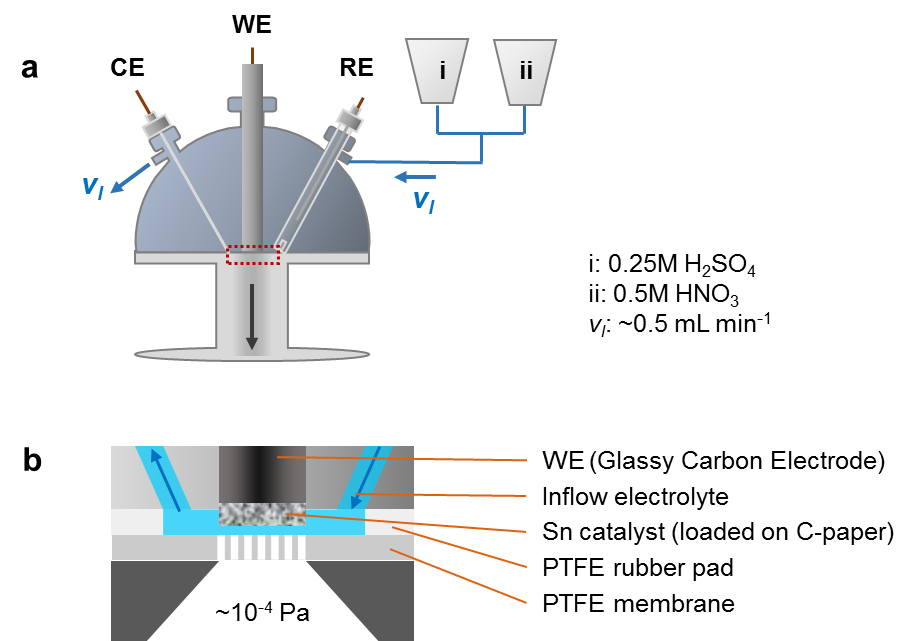


**Supplementary Figure 21** | **Schematic diagram of in-situ DEMS device.** A three-way valve is used to connect 0.25 M H_2_SO_4_ and 0.5 M HNO_3_ solution. The flow rates of the two solutions are controlled by their respective valves. The pH of the two solutions is approximately the same. The purpose of the alternating electrolyte experiment is to separate the spontaneous redox reaction and the electrochemical reduction process. In H_2_SO_4_ solution and at the optimal potential, Sn would catalyse HER. In this process, Sn^II^ can be electroreduced to metallic Sn^0^. In HNO_3_ solution, since Sn and HNO_3_ undergo spontaneous redox reactions and are not affected by electrochemistry, thus the gaseous products detected at this time range are the spontaneous reaction products.

**Supplementary Figure 22 | Intermittent in-situ DEMS device.** DEMS curve of (a) H_2_, (b) NO, (c) NO_2_, (d) NH_2_OH, (e) O_2_.

**Supplementary Note 7**

**Intermittent in situ Raman tests** revealed that, without This nonelectrochemical process is particularly obvious in HNO_3_ solution, whereas it is barely observable in the OA + H₂SO₄ mixture (Supplementary Figures 22−23). This means that in the actual mixed system, there are two key factors that can affect the catalyst valence: (1) an electroreducing process of SnOx under an applied potential and (2) chemical oxidation of Sn to amorphous SnOx in HNO₃-containing solutions.

**Intermittent in situ DEMS** intuitively revealed that the spontaneous oxidation of Sn driven by HNO₃ results in a detectable release of NO (along with a small amount of NO₂), which may be one of the reactants for the subsequent electrochemical N-conversion process (Figure 4f and Supplementary Figure 25-26).

**Supplementary Figure 23** **| Electrochemical in situ XAS over Sn in 0.5 M HNO_3_ electrolyte.** **(a)** In situ Sn K-edge XANES data collected at different potential. The grey line represents the test curve obtained in air. The blue lines represent the test curve obtained in the HNO_3_ electrolyte at −0.3 V vs. SHE. The red lines represent the test curve obtained in the HNO_3_ electrolyte at −1.4 V vs. SHE. **(b)** The in situ valence analysis of Sn based on linear combination analysis of in situ XANES data.

**Supplementary Figure 24** **| Electrochemical in situ XAS over Sn in 0.5 M OA + H_2_SO_4_ electrolyte.** **(a)** In situ Sn K-edge XANES data collected at different potential. The grey line represents the test curve obtained in air. The blue lines represent the test curve obtained in the OA + H_2_SO_4_ electrolyte at −0.3 V vs. SHE. The red lines represent the test curve obtained in the HNO_3_ electrolyte at −1.4 V vs. SHE. **(b)** The in situ valence analysis of Sn based on linear combination analysis of in situ XANES data.

**Supplementary Note 8**

In situ XAS supported this conclusion and quantified the Sn valence in different electrolytes. In a pure HNO_3_ solution, Sn gets slightly oxidized to SnO or SnO_2_, and the content of SnO_x_ is stable at 5~10% (Supplementary Figure 24). However, in the OA and H^+^ electrolytes (which replace HNO_3_ with H_2_SO_4_ while keeping the H^+^ concentration the same), the Sn valence of the initially slighlty oxidized Sn particles gradually decreases over time (Supplementary Figure 24). Thus, in the mixed electrolyte at the optimal potential, the stable components of ~95% Sn^0^ and ~5% SnO should not be in a static state but rather be in a dynamic balance regulated by two influencing factors. In fact, we observe a pronounced oxidation of Sn at less negative potentials, followed by a reduction of Sn under working conditions at –1.4 V vs. SHE (Figure 4d and 4e).

**Supplementary Figure 25** | **Electrochemical test of the standard samples.** Bode plot (**a**) and LSV (**b**) curve for commercial standard samples Sn foil, SnO and SnO_2_ in 0.5 M HNO_3_ + OA mixed electrolyte. The Electrochemical impedance spectroscopy measurement potential is −1 V vs. SHE.

**Supplementary Note 9**

Amorphization alters both valence states and structural configurations. For different valence, the Bode plot of standard sample showed the phase angle and frequency of Sn foil and SnO were similar to our catalyst. Low phase angle and high frequency suggest a faster electron exchange rate.^12^ The LSV curve revealed that *j* of Sn foil was close to the experimental results (Supplementary Figure 4) and significantly larger than those of SnO and SnO_2_ (Supplementary Figure 27)，ruling out the possibility of SnOx as an active site. Based on the results of in situ Raman and in situ XAS, all three catalysts can undergo reconstruction during the electrochemical process, forming Sn/SnOx interfaces, albeit with different bulk compositions. Therefore, from a qualitative analysis perspective, the in situ constructed Sn/SnOx sites may not be the most effective sites. Thus, Sn^0^ may be the main active site, and the amorphous structure may be more conducive to improving the electron transfer rate (Figure 4g).

**Supplementary Figure 26** | **In situ electrochemical quartz crystal microbalance test. (a)** photo of electrochemical quartz crystal microbalance device. We chose gold-plated quartz crystal as the working electrode because the electro-performance of gold is negligible. In situ electrochemical quartz crystal microbalance for crystalline (**b**) and amorphous (**c**) Sn in 0.5 M of HNO_3_, OA, NH_2_OH, GOA and GAO as electrolyte, respectively. Amorphous Sn is obtained by electrocatalysis in 0.5 M OA and HNO_3_ mixed solution for 30 minutes. According to the Sauerbrey equation, negative frequency changes represent increases in electrode mass, corresponding to intercalation or adsorption behaviour of ions/molecules in the electrochemistry process. The loadings of crystalline and amorphous Sn on the electrode are 0.1 and 0.06 mg, respectively. Figure 4h is obtained by calculation based on the data in Supplementary Figure 26.

**Supplementary Note 10**

EQCM enables real-time monitoring of mass changes at electrochemical interfaces through the piezoelectric effect. Here, we use an Au-plated quartz crystal as the cathode. Since Au has poor catalytic properties for synthesis of Gly, the interference caused by the Au film does not need to be considered in this experiment. A certain amount of Sn is deposited on the Au film. The deposition method is the same as that for preparing Sn on C paper (see the Method section). The amount of Sn deposited is about 100 μg according to the resonant frequency change results of the microbalance. According to calculation formula, we can obtain the change of electrode mass during the electrochemical operation.

$$\Delta m=-\frac{\Delta f\times A\times\sqrt{\mu_{q}\times\rho_{q}}}{2f_{q}^{2}}$$

where Δ*m* represents the change mass. Δ*f* represents the resonant frequency change. A represents the area of the gold surface (0.198 cm^2^). *μ_q_* represents the AT-cut quartz constant (2.947 × 10^11^ g cm^−1^ s^−2^). *ρ_q_* represents the quartz crystal density (2.65 g cm^3^). *f_q_* represents the reference frequency (9.00 MHz). Notably, A decrease in frequency corresponds to an increase in electrode mass. The detection limit of Δ*m* is 5 ng.

For this EQCM test (Figure 4h and Supplementary Figure 26), we used 0.5 M of HNO_3_, OA, NH_2_OH, GOA and GAO as electrolyte, respectively. In order to keep the pH of these electrolytes equal, 0.25 M H_2_SO_4_ was added to all solutions except HNO_3_ solution. Notably, we use the resonance frequency of the Sn-modified electrode immersed in the electrolyte as a benchmark. Therefore, Δ*f* = 0 indicates the adsorption state under power-off, rather than no adsorption. The change in resonance frequency Δ*f* (or Δ*m*) represents the average state of the catalytic process (the average value of the adsorption-hydrogenation-desorption process).

For Sn crystals, as the potential shifts negatively, the resonance frequency decreases rapidly in HNO_3_ and OA solutions respectively, corresponding to the increase in electrode mass. When the active sites involved in catalysis reach saturation, the mass change tends to be stable. For the intermediates (NH_2_OH, GOA, GAO), the mass of the electrode hardly changes, suggesting that the adsorption amount does not change significantly with potential.

Differently, for the amorphous Sn catalyst, in addition to the reactant solution, the resonance frequency shows an obvious decrease and increase in the intermediate solution in the GOA/GAO and NH_2_OH electrolytes, respectively. According to the in situ XAS results (Figure 4e), although there is a dynamic balance of redox in the catalyst, the average O-content is stable and low, so the effect of this process on the frequency change can be ignored. The decrease in frequency in GAO and GOA solution means that the adsorption amount increases with the potential negative shift. For NH_2_OH, the increased frequency indicates that the negative shift in potential compared to the off-state is beneficial for the desorption of NH_2_OH.

**Supplementary Figure 27 | Standard IR spectra.** Standard IR spectra of HNO_3_ (**a**), NH_2_OH (**b**), NH_4_^+^ (**c**), OA (**d**), GOA (**e**), GA (**f**), GAO (**g**) and Gly (**h**). The concentration of the standard solution is 0.5 M.

**Supplementary Figure** **28** | **Potential-dependent in situ ATR−IR test of HNO_3_ elelctroreduction and OA electroreduction.** (**a**) Potential-dependent in situ ATR−IR tests over Sn nanoparticles in 0.5 M HNO_3_ electrolyte. (**b**) Potential-dependent in situ ATR−IR tests over Sn nanoparticles in 0.5 M OA + 0.25 M H_2_SO_4_ mixed electrolyte.

**Supplementary Figure 29** | **Time-dependent in situ ATR**−**IR test of HNO_3_ elelctroreduction and OA electroreduction.** (**a**) Time-dependent in situ ATR−IR tests over Sn nanoparticles in 0.5 M HNO_3_ electrolyte. (**b**) Time-dependent in situ ATR−IR tests over Sn nanoparticles in 0.5 M OA + 0.25 M H_2_SO_4_ mixed electrolyte at −1.4 V vs. SHE.


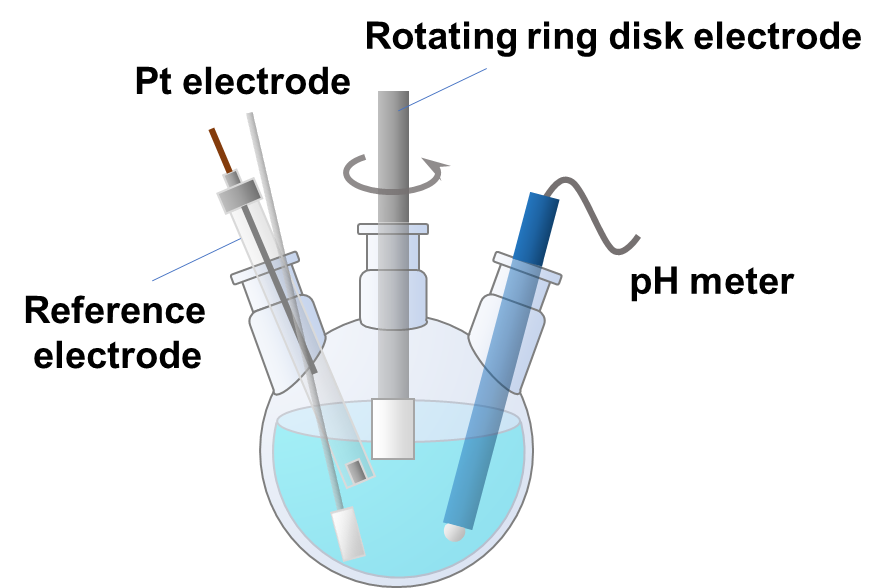


**Supplementary Figure 30 | Local pH Monitoring via RRDE test.** Schematic diagram of the constructed device. The pH meter is used to monitor the H^+^ concentration of the electrolyte. The disk electrode served as the working electrode, and the reduction current of the Pt ring was used to calculate the local pH. The calculation method is based on the results of Supplementary Figure 32.

**Supplementary Figure 31 | RRDE testing for calculation of local pH. (a)** LSV curves of Pt-ring in 0.5 M OA + 0.25 M H_2_SO_4_ solution with different pH and without catalyst on disk. The electrolyte pH can be adjusted by adding 5 M KOH + 0.5 M OA solution. The LSV scan rate was 20 mV s^−1^. The data were acquired with IR correction using the electrochemical workstation without other manual corrections. (**b**) Linear relationship between pH and *E*_SHE_. The data in *E*_SHE_ were selected from the potential corresponding to the current of −1 mA in Supplementary Figure 32a. **(c)** LSV curves of Pt-ring in 0.5 M HNO_3_ solution with different pH and without catalyst on disk. The electrolyte pH can be adjusted by adding 5 M KOH + 0.5 M KNO_3_ solution. The LSV scan rate was 20 mV s^−1^. The data were acquired with IR correction using the electrochemical workstation without other manual corrections. (**b**) Linear relationship between pH and *E*_SHE_. The data in *E*_SHE_ were selected from the potential corresponding to the current of −1 mA in Supplementary Figure 32c. The slopes in Supplementary Figure 32b and 32d is 0.0591 and 0.0600, respectively, which matches the Nernst equation, suggesting that there is a significant Nernst shift in the Pt-ring reduction current, which can be used to represent the local pH.

**Supplementary Figure 32 | RRDE testing in C−intermediate electrolyte. (a, c, e)** LSV curves of Pt-ring in 0.5 M GOA + 0.25 M H_2_SO_4_ (**a**), 0.5 M GA + 0.25 M H_2_SO_4_ (**c**), or 0.5 M NH_3_OHCl solution with different pH and without catalyst on disk. The electrolyte pH can be adjusted by adding 5 M KOH + 0.5 M GOA or 0.5 M GA or 0.5 M NH_3_OHCl solution. The CV scan rate was 20 mV s^−1^. The data were acquired with IR correction using the electrochemical workstation without other manual corrections. (**b, d, f**) Linear relationship between pH and *E*_SHE_. The data in *E*_SHE_ were selected from the potential corresponding to the current of −1 mA in Supplementary Figure 33a, 33c and 33e, respectively. Their slopes are 0.0594, 0.0597 and 0.0605 respectively, close to the pH coefficient in the Nernst equation, eliminating the interference of intermediate on the reduction current.

**Supplementary Note 11**

The principle for selecting the probe molecule/reaction is that it can satisfy the Nernst equation in different pH solutions. In the initial parts of catalyst screening, we found that Pt had almost no reduction performance towards reactants and intermediates. Because, the potentials at low Faradaic current in different electrolytes are similar when the pH is the same, and in solutions with different pH, these potential shifts are also similar. It may be due to that Pt is more likely to undergo HER in an acidic environment, and the HER reaction has a significant Nernstian shift so researchers usually compare the data relative to RHE to facilitate comparison of the catalyst performance in solutions with different pH. The rotation speed of the ring-disk electrode was 1600 rpm, and the results also support our conclusion. Notably, considering that the H^+^ concentration in different pH solutions varies greatly and thus there are kinetic differences, we choose the potential corresponding to the low Faradaic current as a reference. A low Faradaic current (e.g. –1 mA, corresponding to ~5 mA cm^–2^) can indicate the occurrence of electron transfer, and the consumption of H^+^ in the microenvironment is small, thus the system can be approximately regarded as a stable state. By calculating the linear relationship between pH and *E*_SHE_ (Supplementary Figure 32b, 32d, 33b, 33d and 33f), the average value of pH coefficient was be 0.0597, corresponding to the Nernst equation (*E* = *E*^θ^ – *RT*/*nF* × ln[H^+^]).

$\frac{RT}{nF}\ln\left( 10 \right)\approx0.0591$ .

Therefore, in mixed solution, the reduction current of Pt-ring can be selected as a probe to calculate the local pH. The calculation equation is:

*E*_SHE_ = –0.0597pH – 0.0217

It should be noted that this method may have certain limitations, due to the 0.2 mm gap between the Pt ring and the glassy carbon electrode (carried catalyst) of RRDE. Although the tests were conducted at a high rotation speed of 1600 rpm, diffusion limitations may cause the local pH of the Pt ring to be lower than that of the catalyst interface.

**Supplementary Note 12**

Specifically, to establish a calibration curve for local pH measurement, we first conducted experiments under different bulk pH of the electrolyte, which was precisely adjusted by controlled addition of alkaline solution and confirmed using a pH meter. During these measurements, only the Pt-ring electrode was used to record the reduction current, and no catalyst was introduced on the disk electrode. The results show that the reduction current of the Pt-ring can serve as an effective probe for local pH, as it exhibits a clear Nernstian shift with pH and is minimally affected by reactants or possible intermediates (Supplementary Figures 33 and 34), especially under low Faradaic current conditions. Subsequently, RRDE measurements were carried out using an amorphous Sn-modified disk electrode and Pt-ring. Chronoamperometric tests were performed at the optimal potential (−1.4 V vs. SHE) in mixed HNO₃ and OA solutions under various adjusted bulk pH conditions. The RRDE results revealed that the local pH near the electrode surface, calculated from the Pt-ring current, was significantly higher than the bulk pH (Figure 5c). Notably, for the cathode electrolyte in H-cell, the bulk pH of the electrolyte after co-reduction of HNO₃ and OA increased from ~0.45 to ~1.2, and according to Figure 5c, its local pH at the electrode surface can be inferred to be greater than 2.5 under operational conditions. Thus, the organic molecules actually involved in the reaction should be in an ionized state at this elevated pH.

**Supplementary Note 13**

The reason for the in situ local pH change may be that the large applied *j* (~1 A cm^−2^) and the unequal proton−electron transfer numbers (13 protons and 12 electrons transfer for Gly electrosynthesis) lead to local pH alkalinization, especially near the catalyst surface. In the hydrogenation reaction, a large reduction current corresponds to a fast consumption rate of H^+^. When the diffusion rate of H^+^ cannot make up for the consumption rate of H^+^ around the catalyst in time, a pH gradient difference will form on the catalyst surface. For the reaction with different proton-electron transfer numbers, the continuation of the entire reaction process will result in a higher consumption of H^+^ at the cathode than OH^−^ at the anode. Therefore, even if the proton exchange membrane can balance the pH of the anode and cathode, the pH of the entire system will increase as the reaction continues.

OA, GOA and GAO are weak acid. The pH of pure 0.5 M OA, GOA, or GAO solution are 0.81, 0.99 and 1.23, respectively. Among them, 0.5 M OA or GOA solution can be directly prepared. For the GAO solution, equal volumes of 1 M NH_3_OHCl and GOA solutions were mixed to obtain. To neutralize the HCl, an equal amount of KOH needs to be added to balance it.

Taking OA as an example, at the beginning of the reaction, the electrolyte pH is determined by HNO_3_ (K_a_ = 100), and OA tends to be in a molecular state. However, as the reaction proceeds, the consumption of H^+^ is greater than that of OH^−^ at the anode in this all system, even though we are using a proton exchange membrane. Moreover, considering that our experimental current density is industrial-level (~1 A cm^−2^), the limitation of proton transfer rate would lead to a more obvious pH increase of the microenvironment near the catalyst surface. Thus, the pH gradient near the catalyst surface would cause OA to release H^+^ to resist the pH change (similar as a buffer reagent).

The specific ionic/molecular concentrations can be calculated based on Henderson-Hasselbalch equation. Taking OA as an example:

HA ⇌ H+ A^-^

H^+^ ≈ 0.15 M

A^-^ ≈ 0.15 M

HA = 0.5 – 0.15 = 0.35 M

$$Ka=\frac{\left[ H^{+} \right]\times[A^{-}]}{[HA]}$$

So: pKa ≈ 1.19

Before the reaction, the pH of the mixed solution was 0.45, as the main contribution came from the ionization of HNO_3_ (strong electrolyte). According to the Henderson-Hasselbalch equation:

$$pH=pKa+log\frac{[A^{-}]}{[HA]}$$

HA ≈ 0.42 M

A^−^ = 0.5 – 0.42 = 0.08 M

HA represents the molecular form of OA. A^−^ represents the ionic form of OA. H^+^ is the hydrogen ion. We can see that most weak acids exist in molecular form.

Similarly, during the catalytic process, the local pH is 2.5. According to the Henderson-Hasselbalch equation:

$$pH=pKa+log\frac{[A^{-}]}{[HA]}$$

HA = 0.023 M

A^−^ = 0.5 – 0.023 = 0.477 M

Notably, since the in-situ catalytic process is relatively short, the concentration of OA is calculated at 0.5 M. At this point, over 95% of the OA in the microenvironment exists in ionic form.

The existence forms of GOA and GAO are calculated using a similar method. The results showed that the ion concentrations of GOA and GAO during the electrocatalytic process were 0.44 and 0.36 M, respectively. Therefore, the actual form in the microenvironment may be ions rather than molecule.

**Supplementary Figure 33 | Electrochemical impedance spectroscopy.** The Nyquist plot (**a**) and Bode plot (**b**) for the partial and total reactions in Figure 1a. The HER test was carried out in 0.25 M H_2_SO_4_ electrolyte. For the test of R1, R2, R4 and All reaction, the electrolyte is 0.5 M HNO_3_, 0.5 M OA + 0.25 M H_2_SO_4_, 0.5 M NH_2_OH∙HCl + 0.5 M GOA, and 0.5 M HNO_3_ + 0.5 M OA, respectively. Although R_ct_ (radius of the circle) of the total reaction is smaller, its phase angle frequency is the same as that of R2 (single OA reduction), suggesting that R2 has a "bottleneck effect" on the overall reaction.

**Supplementary Figure 34** | **In situ electrochemical quartz crystal microbalance test.** In situ electrochemical quartz crystal microbalance over amorphous Sn at −1.4 V vs. SHE in different electrolyte. The electrolyte is 0.5 M OA + 0.25 M H_2_SO_4_ (**a**), 0.5 M GOA + 0.25 M H_2_SO_4_ (**b**), and 0.5 M GAO + 0.25 M H_2_SO_4_ (**c**), respectively. During the test, 5 M KOH was used to adjust pH. 5 M KOH was slowly added to adjust the pH. The solution pH was detected after vigorous stirring. During the whole process, the electrochemical test was kept running at −1.4 V vs. SHE. The arrows represent the adding KOH with vigorous stirring. The solution pH after adding KOH is marked on the arrow. The loadings of amorphous Sn on the electrode are 0.1 mg. Notely, among this testing, there is no HNO_3_ in the electrolyte. According to the above conclusions (Figure 4), there is no catalyst reconstruction behavior.

**Supplementary Figure 35** | **DFT calculations.** Adsorption energy and optimized geometries of molecules on crystallized Sn (101) surface, such as OA (**a**), OA^−^ (**b**), GOA (**c**) and GOA^−^ (**d**). Positive adsorption energy indicates thermodynamically unfavourable, which imply that the ionized state of OA and GOA molecules can not make it more susceptible to adsorption.

**Supplementary Figure 36** | **Photo of the device.** The operation photo of the scale up flow cell. The operating current is 10 A. the electrolyzer potential is ~4.6 V. The flow rate of the electrolyte is 100 rpm (corresponding to 30 mL min^−1^).


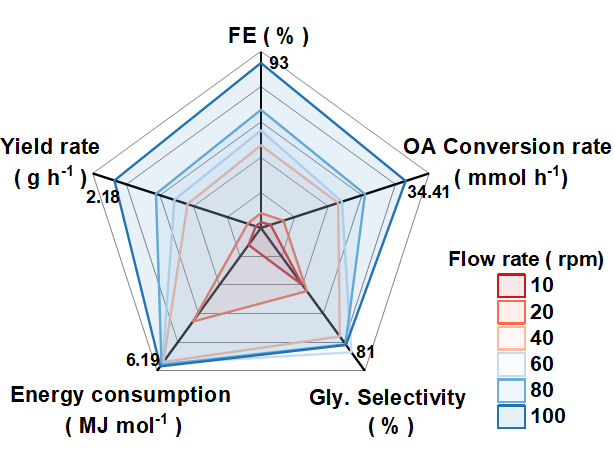


**Supplementary Figure 37 | Scale-up experiments for the electrosynthesis of Gly**. Summary of the electrocatalytic performance of flow devices. Unless otherwise stated, the cathode electrolyte is 0.5 M HNO_3_ + 0.5 M OA, and the volume is 10 mL. The electrolyte at the anode is 0.25 M H_2_SO_4_. The experimental current is 10 A. The electrode area of the ​​cathode and anode is 10 cm^2^.


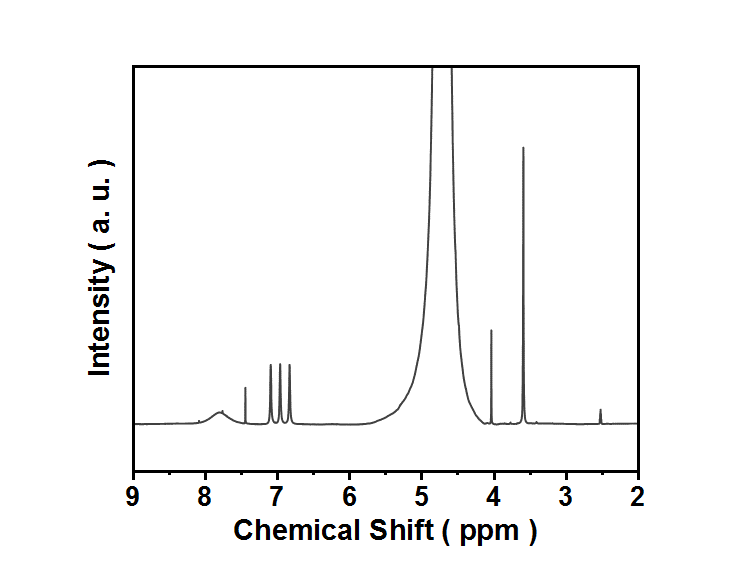


**Supplementary Figure 38 | ^1^H NMR spectra**. ^1^H NMR spectra of the flow cell electrolyte after 15 min electro-testing. The electrolyte is 5 mL of 1 M OA and 7 mL of 1 M HNO_3_ mixed solution.


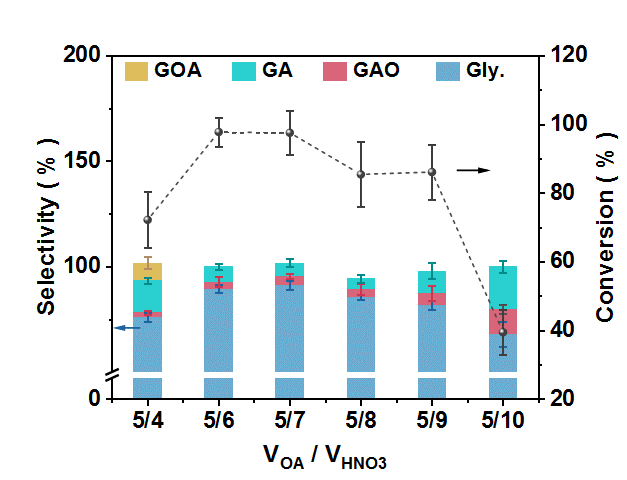


**Supplementary Figure 39 | Electro-testing of different ratios of reactants**. OA conversion and Gly selectivity for flow devices. The experimental current is 10 A. The ratio of reactants can be changed by adding different volumes of HNO_3_ (1 M) to 5 mL of OA solution (1 M).

**Supplementary References**

1 Wang, H. *et al.* Imidazolium radical-mediated electron transfer enhances electrochemical C–N coupling for glycine synthesis. *Nat. Synth.*, (2025). DOI: https://doi.org/10.1038/s44160-025-00892-7.

1 Cheng, Y. *et al.* Highly efficient electrosynthesis of glycine over an atomically dispersed iron catalyst. *J. Am. Chem. Soc.* **146**, 10084-10092 (2024).

2 Kim, J. E. *et al.* Electrochemical synthesis of glycine from oxalic acid and nitrate. *Angew. Chem.* **133**, 22114-22122 (2021).

3 Liao, P. *et al.* Cu−Bi bimetallic catalysts derived from metal–organic Framework arrays on copper foam for efficient glycine electrosynthesis. *Angew. Chem. Inter. Ed.* **64**, e202417130 (2025).

4 Zhu, Z. *et al.* Highly Efficient Synthesis of α‐Amino Acids via Electrocatalytic C‐N Coupling Reaction Over an Atomically Dispersed Iron Loaded Defective TiO2. *Adv. Mater.* **37**, 2409864 (2025).

5 Xu, Y.-Z., Abbott, D. F., Poon, L. N. & Mougel, V. Two-step tandem electrochemical conversion of oxalic acid and nitrate to glycine. *EES Catal.* (2025). DOI: 10.1039/d5ey00016e.

6 Liu, S. et al. Electrochemical lattice engineering of bismuthene for selective glycine synthesis. *Adv. Mater.*, **37**, 2500843 (2025).

8 Wang, K., Li, P. & Zhang, B. Industrial-grade electrocatalytic synthesis of glycine from oxalic acid and nitrate using a porous PbSnBi catalyst. *Appl. Catal. B Environ Energy*, **361**, 124653 (2025).

9 Takashima, T., Wakatsuki, K. & Irie, H. Electrochemical synthesis of glycine from oxalate and nitrate ions using titanium oxide electrode. *Appl. Catal. B Environ Energy,* **371**, 125272 (2025).

10 Li, M. *et al.* Electrosynthesis of amino acids from NO and α-keto acids using two decoupled flow reactors. *Nat. Catal.* **6**, 906-915 (2023).

11 Xian, J. *et al.* Electrocatalytic Synthesis of Essential Amino Acids from Nitric Oxide Using Atomically Dispersed Fe on N‐doped Carbon. *Angew. Chem. Inter. Ed.* **62**, e202304007 (2023).

12 Chi, H. *et al.* Electrosynthesis of ethylene glycol from biomass glycerol. *Nat. Commun.* **16**, 979 (2025).
