# Supplementary material for: Sn catalyst reconstruction and microenvironment modulation for efficient amino acid electrosynthesis via C–N coupling
Source: Nat Commun. 2026 Apr 21;17:3614. doi: 10.1038/s41467-026-71694-4 (PMC13100173; doi:10.1038/s41467-026-71694-4)
Supplement: Supplementary file 2 — Description of Additional Supplementary Files [file 41467_2026_71694_MOESM2_ESM.pdf]

Description of Additional Supplementary Files

File Name: Supplementary Data 1

Description: This file refers to the geometry after molecular dynamics (MD) simulations with non-dissociated H2X ($C_2H_2O_4$) adsorbed. It was carried out to generate well-distributed geometry for following dissociation study. It is a periodic slab cell (a=9.180 Å, b=12.982 Å, c=36.000 Å, α=β=γ=90°) and top Sn-layer shows slight distortion due to H2X adsorption. MD has been carried out with time step of 1 fs, running for 2 ps using ab initial approach under the scheme of spin-polarized DFT, together with PBE functional and energy converged to 10^{-4} eV and force to 10^{-4} eV/Å (more details as listed in the DFT method).

File Name: Supplementary Data 2

Description: This file refers to the initial geometry for MD simulations for clean crystalized Sn surface covered by water layers. It was carried out to generate well-distributed geometry for following H2A and H2X adsorption and dissociation study. It is a periodic slab cell (a=9.180 Å, b=12.982 Å, c=36.000 Å, α=β=γ=90°) and top Sn-layer shows slight distortion due to H2X adsorption. MD has been carried out with time step of 1 fs, running for 2 ps using ab initial approach under the scheme of spin-polarized DFT, together with PBE functional and energy converged to 10^{-4} eV and force to 10^{-4} eV/Å (more details as listed in the DFT method).

File Name: upplementary Data 3

Description: This file refers to the initial geometry for MD simulations for clean amorphous Sn surface covered by water layers. It was generated using amorphous cell first and followed by loading 40 water molecules. Such geometry was used as initial configuration to investigate whether amorphous surface will distort further when water solution has been introduced.

File Name: upplementary Data 4

Description: This file was obtained using a-Sn-MD-IS.cif as starting point, followed by MD simulation. Specifically, MD has been carried out with time step of 1 fs, running for 2 ps using ab initial approach under the scheme of spin-polarized DFT, together with PBE functional and

energy converged to 10^{-4} eV and force to 10^{-4} eV/Å.

File Name: Supplementary Data 5

Description: This file refers to the optimized geometries with crystalized Sn surface (6 layers, Sn_{48}) with non-dissociated H2X ($C_2H_2O_4$) adsorbed, covered by water layers (40 H_2O molecules). Specifically, it is a periodic slab cell (a=9.180 Å, b=12.982 Å, c=36.000 Å, α=β=γ=90°) and top Sn-layer shows slight distortion due to H2X adsorption.

File Name: Supplementary Data 6

Description: This file refers to the optimized geometries with crystalized Sn surface (6 layers, Sn_{48}) with dissociated H2X, labelled as HX with single proton released into water layers. The structure is a periodic slab cell (a=9.180 Å, b=12.982 Å, c=36.000 Å, α=β=γ=90°) and top Sn-layer shows slight distortion due to HX adsorption.

File Name: Supplementary Data 7

Description: This file refers to the optimized geometries with crystalized Sn surface (6 layers, Sn_{48}) with non-dissociated H2A ($C_2H_2O_3$) adsorbed, covered by water layers (40 H_2O molecules). Specifically, it is a periodic slab cell (a=9.180 Å, b=12.982 Å, c=36.000 Å, α=β=γ=90°) and top Sn-layer shows slight distortion due to H2X adsorption.

File Name: Supplementary Data 8

Description: This file refers to the optimized geometries with crystalized Sn surface (6 layers, Sn_{48}) with dissociated H2A, labelled as HA with single proton released into water layers. The structure is a periodic slab cell (a=9.180 Å, b=12.982 Å, c=36.000 Å, α=β=γ=90°) and top Sn-layer shows slight distortion due to HX adsorption.

File Name: Supplementary Data 9

Description: This file refers to the optimized geometry with amorphous Sn surface (Sn_{44}) with non-dissociated H2X ($C_2H_2O_4$) adsorbed, covered by water layers (40 H_2O molecules). Specifically, it is a periodic slab cell (a=9.099 Å, b=12.868 Å, c=35.683 Å, α=β=γ=90°).

File Name: Supplementary Data 10

Description: This file refers to the optimized geometry with amorphous Sn surface (Sn_{44}) with dissociated H2X, shown as HX (adsorbed on the surface) and another proton released into water layers. The structure is a periodic slab cell (a=8.872 Å, b=12.547 Å, c=34.792 Å, α=β=γ=90°).

File Name: Supplementary Data 11

Description: This file refers to the optimized geometry with amorphous Sn surface (Sn_{44}) with non-dissociated H2A ($C_2H_2O_3$) adsorbed, covered by water layers (40 H_2O molecules). Specifically, it is a periodic slab cell (a=9.180 Å, b=12.982 Å, c=36.000 Å, α=β=γ=90°).

File Name: upplementary Data 12

Description: This file refers to the optimized geometry with amorphous Sn surface (Sn_{44}) with dissociated H2A, labelled as HA with single proton released into water layers. The structure is a periodic slab cell (a=9.180 Å, b=12.982 Å, c=36.000 Å, α=β=γ=90°).
